# Bacteria pathogens drive host colonic epithelial cell promoter hypermethylation of tumor suppressor genes in colorectal cancer

**DOI:** 10.1186/s40168-020-00847-4

**Published:** 2020-07-16

**Authors:** Xiaoxuan Xia, William Ka Kei Wu, Sunny Hei Wong, Dabin Liu, Thomas Ngai Yeung Kwong, Geicho Nakatsu, Pearlly S. Yan, Yu-Ming Chuang, Michael Wing-Yan Chan, Olabisi Oluwabukola Coker, Zigui Chen, Yun Kit Yeoh, Liuyang Zhao, Xiansong Wang, Wing Yin Cheng, Matthew Tak Vai Chan, Paul Kay Sheung Chan, Joseph Jao Yiu Sung, Maggie Haitian Wang, Jun Yu

**Affiliations:** 1 Division of Biostatistics, Centre for Clinical Research and Biostatistics, JC School of Public Health and Primary Care, The Chinese University of Hong Kong, Shatin, N.T. Hong Kong, Special Administrative Region of China; 2 Department of Anaesthesia and Intensive Care, The Chinese University of Hong Kong, Shatin, N.T., Hong Kong, Special Administrative Region of China; 3 Li Ka Shing Institute of Health Sciences, The Chinese University of Hong Kong, Shatin, N.T., Hong Kong, Special Administrative Region of China; 4 State Key Laboratory of Digestive Diseases, Institute of Digestive Diseases, CUHK-Shenzhen Research Institute, The Chinese University of Hong Kong, Shatin, N.T., Hong Kong, Special Administrative Region of China; 5 Division of Hematology, Department of Internal Medicine, The Ohio State University, Columbus, OH USA; 6 Department of Biomedical Sciences, National Chung Cheng University, Chia-Yi, Taiwan, Republic of China; 7 Department of Microbiology, The Chinese University of Hong Kong, Shatin, N.T., Hong Kong, Special Administrative Region of China; 8 Department of Medicine and Therapeutics, The Chinese University of Hong Kong, Shatin, N.T., Hong Kong, Special Administrative Region of China

**Keywords:** Colorectal cancer, Microbiota, DNA methylation, MBDCap-Seq, DNMT

## Abstract

**Background:**

Altered microbiome composition and aberrant promoter hypermethylation of tumor suppressor genes (TSGs) are two important hallmarks of colorectal cancer (CRC). Here we performed concurrent 16S rRNA gene sequencing and methyl-CpG binding domain-based capture sequencing in 33 tissue biopsies (5 normal colonic mucosa tissues, 4 pairs of adenoma and adenoma-adjacent tissues, and 10 pairs of CRC and CRC-adjacent tissues) to identify significant associations between TSG promoter hypermethylation and CRC-associated bacteria, followed by functional validation of the methylation-associated bacteria.

**Results:**

*Fusobacterium nucleatum* and *Hungatella hathewayi* were identified as the top two methylation-regulating bacteria. Targeted analysis on *bona fide* TSGs revealed that *H. hathewayi* and *Streptococcus* spp*.* significantly correlated with *CDX2* and *MLH1* promoter hypermethylation, respectively. Mechanistic validation with cell-line and animal models revealed that *F. nucleatum* and *H. hathewayi* upregulated DNA methyltransferase. *H. hathewayi* inoculation also promoted colonic epithelial cell proliferation in germ-free and conventional mice.

**Conclusion:**

Our integrative analysis revealed previously unknown epigenetic regulation of TSGs in host cells through inducing DNA methyltransferase by *F. nucleatum* and *H. hathewayi*, and established the latter as CRC-promoting bacteria.

Video abstract.

## Introduction

Colorectal cancer (CRC), the fourth most common cancer and the fifth leading cause of cancer death globally [1], occurs as a result of intricate interactions between host and environmental factors, among which gut dysbiosis is strongly associated with disease occurrence [2, 3]. In particular, metagenomic studies by us and other investigators have demonstrated compositional alterations of stool and mucosal microbiomes across stages of colorectal tumorigenesis that are characterized by ectopic overgrowth of oral pathogens and depletion of multiple commensal species [4–7]. Subsequent functional studies of CRC-enriched species have further led to identification of pivotal mutagenic or tumor-promoting bacteria (e.g., *pks*-positive *Escherichia coli*, *Fusobacterium nucleatum*, *Peptostreptococcus anaerobius*) that play direct causative roles in CRC [8–10]. Transplantation of stool from CRC patients into germ-free and conventional mice also confirmed the causal link of gut microbes to CRC development [11]. These evidences collectively support a key functional role of bacteria in CRC pathogenesis.

Both genetic and epigenetic pathways are involved in CRC development, in which the latter aberrantly silences tumor suppressor genes (TSGs) via multiple mechanisms, including promoter hypermethylation and histone modifications [12]. DNA methylation involves the covalent addition of a methyl group to the 5-carbon of the cytosine ring within CG dinucleotides of gene promoters by DNA methyltransferases (DNMTs) to form 5-methylcytosine (5-mC). In this regard, gene-specific promoter hypermethylation has been widely reported in CRC with many methylated genes functionally verified as *bona fide* TSGs (e.g., *APC*, *MLH1*, *PTEN*, *RUNX3*, *CDX1*/*2*) [13]. Nevertheless, the upstream molecular events regulating DNA methylation in relation to etiological factors of CRC remain largely elusive.

Sporadic studies have attempted to investigate the effects of the gut microbiome or specific microorganisms on DNA methylation in colonic epithelial cells. In a porcine model, early microbial colonization was found to affect DNA methylation of genes related to immune response, phagocytosis, endothelial homeostasis, and tissue metabolism [14]. Exposure to probiotics or pathogenic bacteria in the context of necrotizing enterocolitis that primarily affects preterm infants has also been shown to induce differential DNA methylation patterns in colonic epithelial cells [15]. Moreover, the gut microbiome was found to be essential for guiding epigenetic development of intestinal stem cells during early development [16]. These studies suggest the existence of an important linkage between the gut microbiome and the host-cell methylome. The microbiome-methylome axis in CRC, however, has not been systematically investigated. Here, we performed concurrent profiling of the methylome and mucosal microbiome in CRC patients to investigate associations between methylation signatures and microbial abundances. We demonstrated that CRC-associated bacteria modulated DNA methylation patterns in colonic epithelial cells to drive intestinal tumorigenesis.

## Methods

### Patient recruitment and informed consent

We enrolled individuals who had undergone standardized colonoscopic examinations at the Prince of Wales Hospital of the Chinese University of Hong Kong. Written informed consents were obtained from subjects or their authorized representatives. Eligibility criteria for colonoscopy included (1) age 50–70 years; (2) absence of existing or previous CRC symptoms, such as hematochezia, tarry stool, change in bowel habit in the past 4 weeks or a weight loss of > 5 kg in the past 6 months; and (3) not having received any CRC screening tests in the past 5 years. Biopsies were snap-frozen in cryovials immediately after polypectomy and stored at − 80 °C until DNA extraction. Adjacent normal tissues were taken at least 4 cm away from lesions. Colorectal mucosae were obtained using cold biopsy forceps separately for lesions and lesions-adjacent tissues to avoid cross-contamination between samples.

### Methylated-DNA capture sequencing

Genomic DNA samples of colonic mucosal biopsies were obtained with informed consent as previously described [5]. Methylated DNA was enriched using the MethylMiner^TM^ kit (cat. ME10025, Invitrogen, US) [17]. Briefly, DNA was sonicated and incubated at a ratio of 1:3.5 with human MBD2-biotin conjugated to Dynabeads® M-280 Streptavidin. Methylated DNA was purified from magnetic bead complex by performing 3 washes with Bind/Wash Buffer to remove the uncaptured fraction of DNA. Captured DNA was eluted with 1 M NaCl and precipitated in ethanol. Single-end 50-bp Illumina libraries were prepared for enriched DNA samples, which were subjected to sequencing on the Illumina Genome Analyzer IIx according to manufacturer’s protocol.

### MBDCap sequence quality control

Raw sequences were quality-trimmed using Trimmomatic (v0.36) in maximal information mode [18], which was configured at 99.9% strictness to find and trim any bases having Phred quality scores < 30. Sequence reads were simultaneously screened, flagged for, and trimmed of Illumina adapter sequences; those that had fewer than 35 bases were finally discarded prior to downstream processing. We decontaminated post-Trimmomatic sequences using the Burrows-Wheeler Aligner with default parameters of maximal exact match algorithm (bwa-mem, v0.7.17-r1188) by aligning them against an indexed database consisting of 6877 bacterial plasmids, complete genomes of 8117 mitochondria, and 2116 plastomes as well as 4522 EmVec and 6093 UniVec sequencing vectors (NCBI RefSeq accessed on May 12, 2016) [19]. Sequences were mapped to dust-masked GENCODE human genome (GRCh38.p12) with bwa-mem aligner for detection and removal of optical and PCR duplicates using the MarkDuplicates module in the Picard toolkit (v2.14.0) [20].

### Statistical inference and analysis of genome-wide DNA methylome

MBDCap sequence enrichment signals were modeled and transformed to infer absolute levels of DNA methylation using the QSEA R/Bioconductor package for which we performed parametrization and provided external calibration datasets [21]. Average single-end sequencing fragment length was estimated using the makeTagDirectory command in the HOMER bioinformatic suite [22]. An optimal size of sliding window for aligned reference coordinates in each sample BED file was selected by defining the lowest Akaike’s information criterion (AIC) of read count density function across an incremental range of genomic segment sizes [23]. A custom Biostrings-based genome data infrastructure was created for the GENCODE human genome assemblies using the BSgenome R/Bioconductor package and imported into qseaSet object. We defined copy number variations (CNVs) across 1 megabase segment with the built-in HMMcopy function to adjust for effects on read count normalization as cancer samples were included in our dataset [24]. A genomic segment was considered CpG-free if the pattern density was lower than 0.001%, which was used to offset background reads. To calibrate parameters of CpG enrichment function, we obtained publicly available Illumina Infinium Methylation 450K BeadChip data. This calibration data included methylation profiles of COAD and READ projects from the GDC Data Portal of the Cancer Genome Atlas (TCGA) as well as of independent projects of a similar study design (GSE48684 and GSE53051) from the Gene Expression Omnibus (GEO) [25, 26]. These datasets were preprocessed through the Minfi R/Bioconductor package and normalized separately via the SWAN algorithm [27, 28]. Potential effects of technical variation (TCGA plate IDs) were removed before combining normalized datasets to correct for study batches using the ComBat algorithm as implemented in the sva R/Bioconductor package [29]. We filtered Infinium 450K CpG probes that had average beta scores of < 0.9 and standard deviations of > 0.05 for a tissue phenotype group [21]; those that met these criteria were assigned to chromosomal coordinates to calculate average beta score for each 200-bp genome bin across primary human genome assemblies [30]. As a result, we identified 55,341 genomic regions that had low variability in methylation over an integrated set of TCGA-GEO samples for calibration of enrichment profiles. To enable comprehensive genomic feature-guided methylome profiling and analysis, we imported GENCODE gff3 annotation file and unmasked CpG island information from the UCSC Golden Path services as well as genomic coordinates of FANTOM5-CAGE enhancers and miRNA promoters [31, 32]. Differentially methylated regions (DMRs) between tissue phenotypes and between microbiome subtypes were detected using the limma R/Bioconductor package [33], and robust pairwise fold change (pfc) was determined by calculating the average of pfc values that fell within an interquartile range [34]. Stratified hierarchical clustering was performed according to the Ward’s minimum-variance method using Euclidian distance-based sample dissimilarity matrix; methylotypes were defined by a consensus analysis employing 30 indices for cluster optimization as implemented in NbClust R/CRAN package.

### RNA extraction and real-time PCR

Total RNA was extracted from cells and tissues using TRIzol Reagent (Molecular Research Center Inc.). cDNA was synthesized from 1 μg of total RNA using Transcriptor Reverse Transcriptase (Roche). Real-time PCR was performed using SYBR Green master mixture (Roche) on LightCycler 480 Instrument. Each sample was tested in triplicate. ΔΔC_T_ method was applied to determine the fold change in gene expression. ΔC_T_ method was applied to determine the relative expression of corresponding genes.

### Western blots

Total protein was separated by sodium dodecyl sulfate-polyacrylamide gel electrophoresis (SDS-PAGE). The proteins in SDS-PAGE were transferred onto nitrocellulose membranes (GE Healthcare). The membrane was incubated with primary antibodies overnight at 4 °C, and then with secondary antibody at room temperature for 1 h. Proteins of interest were visualized using ECL Plus Western blotting Detection Reagents (GE Healthcare).

### Global DNA methylation (5-mC) level analysis

Two hundred nanograms of genome DNA were harvested, and DNA methylation levels were detected by MethylFlash Global DNA Methylation (5-mC) ELISA Easy Kit (p-1030, EpiGentek) according to the manufacturer’s instructions. All experiments were conducted three times in triplicate. Results are shown as mean ± SEM.

### DNMT activity assay

Twenty micrograms of nuclear proteins were harvested, and DNMT activities were detected by DNMT assay kits (ab113469 for DNMT1, ab113470 for DNMT3A, and ab113471 for DNMT3B, Abcam) according to the manufacturer’s instructions. All experiments were conducted three times in triplicate. Results are shown as mean ± SEM.

### Animal experiments

Conventional C57BL/6 mice were bred in the Laboratory Animal Services Centre at the Prince of Wales Hospital, The Chinese University of Hong Kong. Male C57BL/6 mice (6 weeks of age) were treated with a cocktail of broad-spectrum antibiotics, including ampicillin (0.2 g/L), metronidazole (0.2 g/L), neomycin (0.2 g/L), and vancomycin (0.1 g/L), in their drinking water for 1 week, followed by a single intraperitoneal injection of azoxymethane (AOM, 10 mg/kg). One-week post AOM injection, mice were gavaged with 1 × 10^8^ c.f.u. of *H*. *hathewayi* every 2 days for 6 weeks. For germ-free mice experiments, KunMing mice were bred at the Department of Laboratory Animal Science at the Third Military Medical University in Chongqing, China. Adult germ-free mice (8 weeks of age) were gavaged with *H*. *hathewayi* 1 × 10^8^ c.f.u. of *H*. *hathewayi* every 2 days for 20 weeks. At the end of each experiment, mice were anaesthetized with isoflurane followed by cardiac puncture to collect arterial blood, and cervical dislocation before dissecting for tissue collection.

### Mouse tissue collection and histological evaluation

After sacrifice, the whole colon of the mice was flushed with phosphate-buffered saline (PBS) and excised longitudinally. The colon was first examined for erosion and abnormality, and then divided into 3 sections (proximal, middle, and distal). Half of each colon section was fixed in 10% formalin, processed and paraffin-embedded for histologic examination. The remaining half of each colon section was snap-frozen and kept at − 80 °C for subsequent molecular analyses. Four 5-μm tissue sections from each colonic segment were stained with hematoxylin and eosin. The Ki-67 expression of the paraffin-embedded tissues was analyzed by immunohistochemistry. The proportion of Ki-67 positive cells was determined by counting immunostain-positive cells, as a percentage to the total number of nuclei in the field.

### Statistical methods

Differentiated methylation and microbiome segments were tested by the Wilcoxon signed rank test for paired samples, and the Mann-Whitney *U* test for independent samples. Zero-inflated negative binomial regression was used to test for the interaction between the microbiome species count and methylation gene expression, implemented by R package *pscl*. The analysis was repeated using an alternative Spearman’s rank-order correlation test to check for consistency. Heat maps were used to visualize differential abundances in the methylome and microbiome across different CRC phenotypes. All analyses were conducted in R.

## Results

### Parallel methylome and microbiome profiling in colonic adenomas, CRC, and corresponding adjacent tissues

**Fig. 1:**
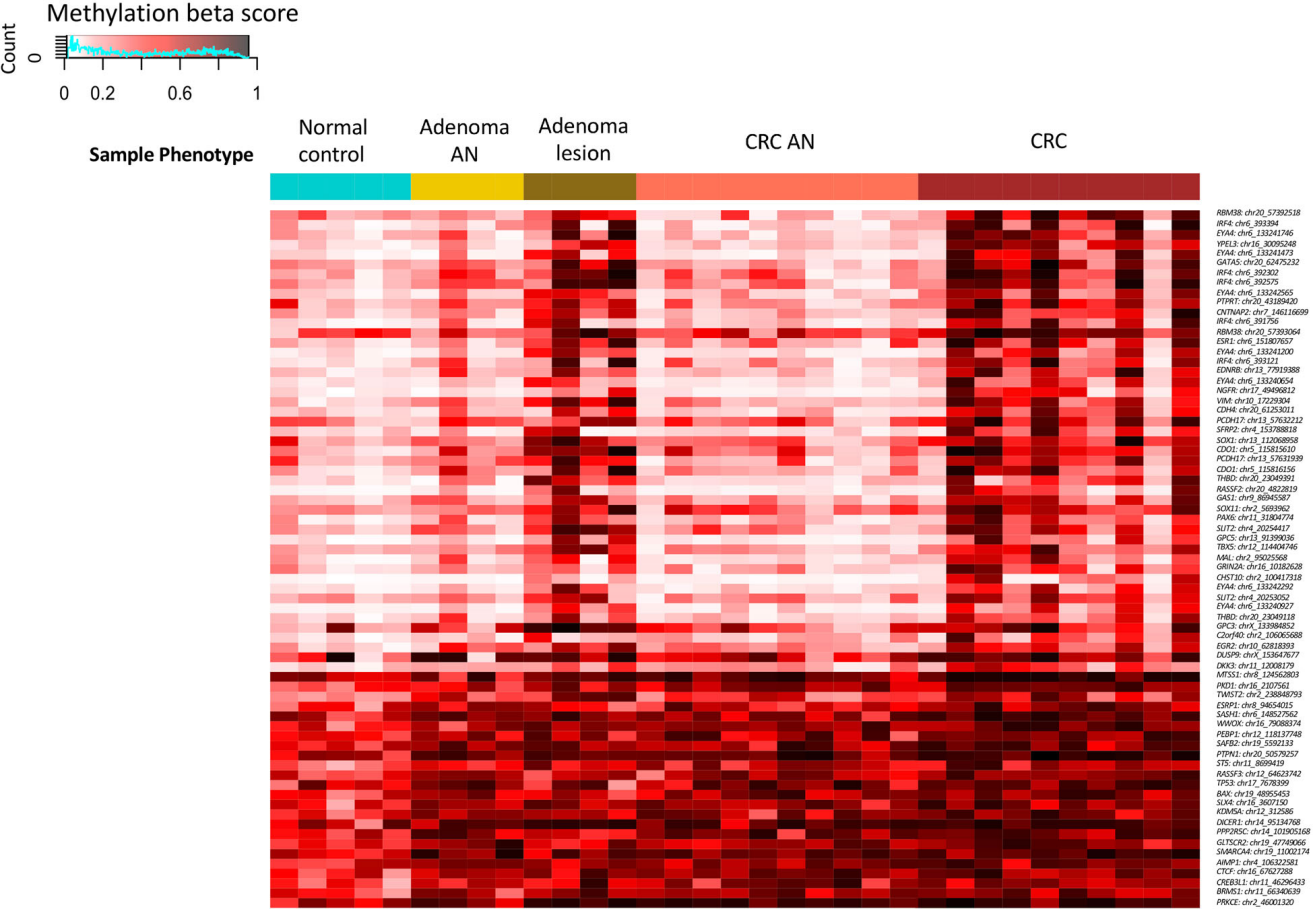
Tumor suppressor genes (TSGs) hypermethylation in adenomas and CRC. Heatmap of TSG promoter regions showed pervasive hypermethylation in adenomas and CRC samples. The methylation segments shown are the genes with differential methylation between any two phenotype groups (*P* < 0.005). AN, adjacent

Genome-wide DNA methylation profiling by methyl-CpG binding domain-based capture sequencing (MBDCap-Seq) was performed in thirty-three tissue biopsies from five normal colonic mucosa tissues, four pairs of adenoma and adenoma-adjacent tissues, and ten pairs of CRC and CRC-adjacent tissues. Genome-wide coverage of MBDCap sequence reads (Figure S1) and genome-wide distribution of differentially methylated regions across phenotypes (Figure S2) were first determined followed by targeted analysis of promoters of known TSGs (as curated by the TSGene database) [35]. Among the differentially methylated segments (*p* < 0.005, Wilcoxon signed rank test for paired sample or Mann-Whitney *U* test for unpaired sample comparison), the majority were hypermethylated in tumor samples as compared to corresponding adjacent controls. Moreover, one fifth of the CpG sites showed a progressive trend of methylation alterations along the adenoma-carcinoma sequence (Fig. 1).

Parallel microbiome profiling from the same set of samples was performed by 16S rRNA gene amplicon sequencing study (Figure S3) [5]. Original abundances of taxa were used to perform subsequent analysis as recommended by Weiss et al. [36]. Paired analysis of adenoma, CRC, and their adjacent tissues showed that *F. nucleatum* (fold change = 22.34, *p* = 0.009), *Hungatella hathewayi* (fold change = 9.67, *p* = 0.042), and *Parvimonas* spp. (fold change = 5.78, *p* = 0.009) were the top three bacteria that showed significant enrichment in tumor tissues compared to corresponding adjacent controls.

### *F. nucleatum* and *H. hathewayi* showed significantly stronger interactions with methylation of TSG promoters

**Fig. 2:**
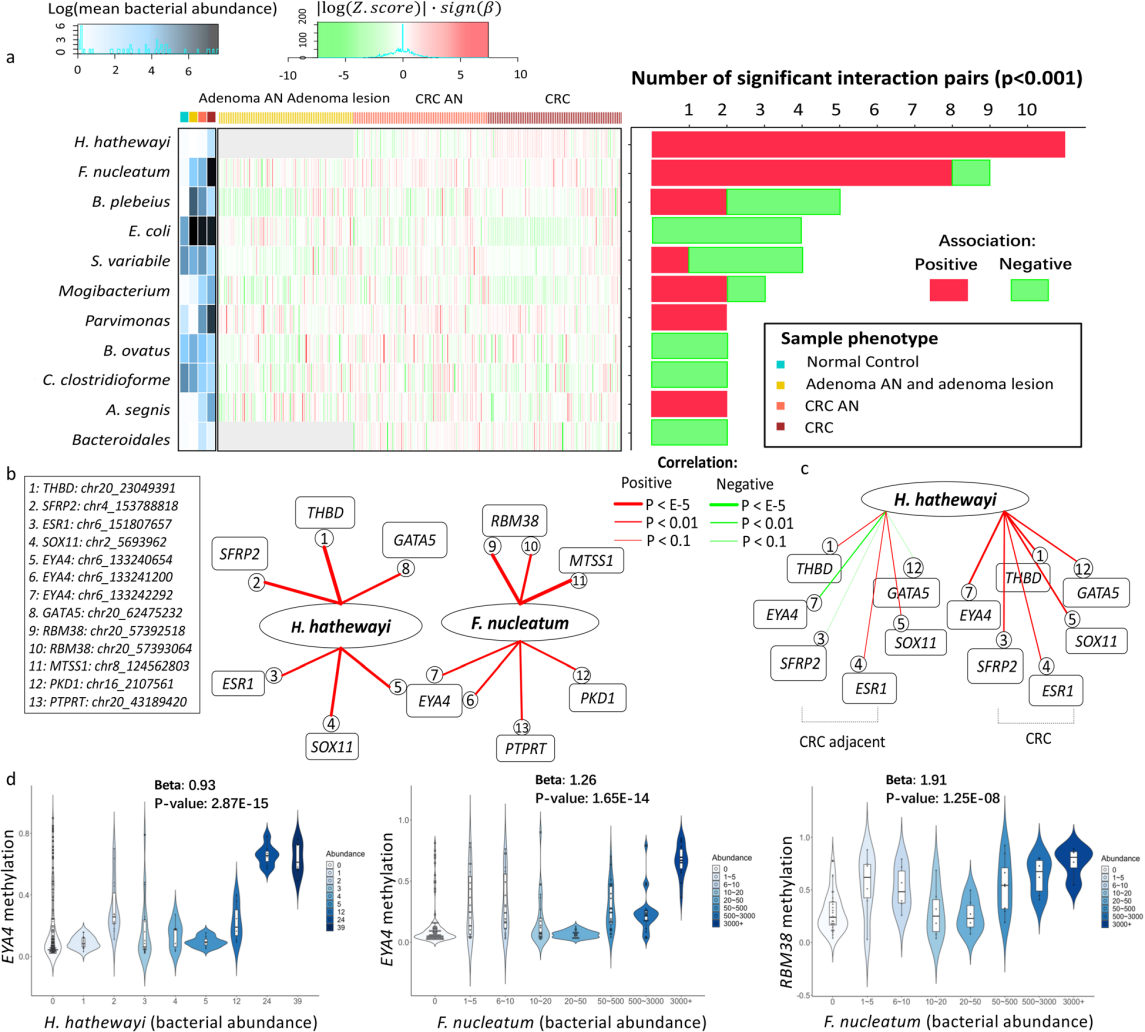
Interactions between CRC-associated bacteria and TSG methylation. **a** Heatmap of interaction effect coefficients between bacteria abundance and TSG methylation. The interaction was calculated in three phenotype groups respectively: adenoma-adjacent (AN) tissues plus adenomas, CRC-AN tissues, and CRC. Horizontally, *H. hathewayi* and *F. nucleatum* showed increased positive associations as tumor progressed. The gray color denotes undefined interaction due to empty count of bacteria in a phenotype group. Vertically, the bacteria are ranked according to the quantity of interactions they involved in. Bacteria mean abundance heatmap were appended to the left of the interaction heatmap. **b** Significant interaction networks involving *H. hathewayi* and *F. nucleatum* in the non-normal samples were shown. **c** Interactions involving *H. hathewayi* in the CRC-AN and CRC groups were depicted separately in CRC and CRC-adjacent groups. Red: positive association; Green: negative association. The direction of interaction effects was significantly positive in the CRC group. **d** Violin plot of TSG methylation versus bacteria abundance. The methylation levels of gene *EYA4* and *RBM38* increased as *H. hathewayi* and *F. nucleatum* abundances increased

Next, we evaluated whether the abundances of mucosal microorganisms identified above were associated with differentially methylated TSGs. *F. nucleatum* and *H. hathewayi* had the highest number of significant associations with TSG promoter methylation across stages of CRC development (*p* < 0.001, zero-inflated negative binomial regression) (Fig. 2a). Furthermore, the interactions involving these two bacteria were predominantly positive in direction, with higher abundances correlating with higher levels of TSG promoter methylation. *H. hathewayi* was associated with methylation of TSGs including *SOX11*, *THBD*, *SFRP2*, *GATA5*, and *ESR1*, whereas *F. nucleatum* was associated with methylation of *MTSS1*, *RBM38*, *PKD1*, and *PTPRT* (Fig. 2b; Table S1). We also observed that, while associations between bacteria and TSG promoter methylation status were composed of a mixture of positive and negative correlations in the normal colonic mucosa samples, the direction of correlations was uniformly positive in CRC samples (Fig. 2c). These results suggested that enrichment of *H. hathewayi* and *F. nucleatum* were associated with pervasive TSG-promoter methylation. Statistical replication was performed using an alternative non-parametric test by the Spearman rank correlation. Consistent with the results from zero-inflated negative binomial regression, *EYA4* promoter-wide methylation was positively associated with both *H. hathewayi* (negative binomial *p* = 2.87E-15, Spearman *p* = 2.21E-4) and *F. nucleatum* (negative binomial *p* = 1.65E−14, Spearman *p* = 2.15E−12) as displayed in the violin plots (Fig. 2d).

### Bacteria interactions with *bona fide* TSGs in CRC

**Fig. 3:**
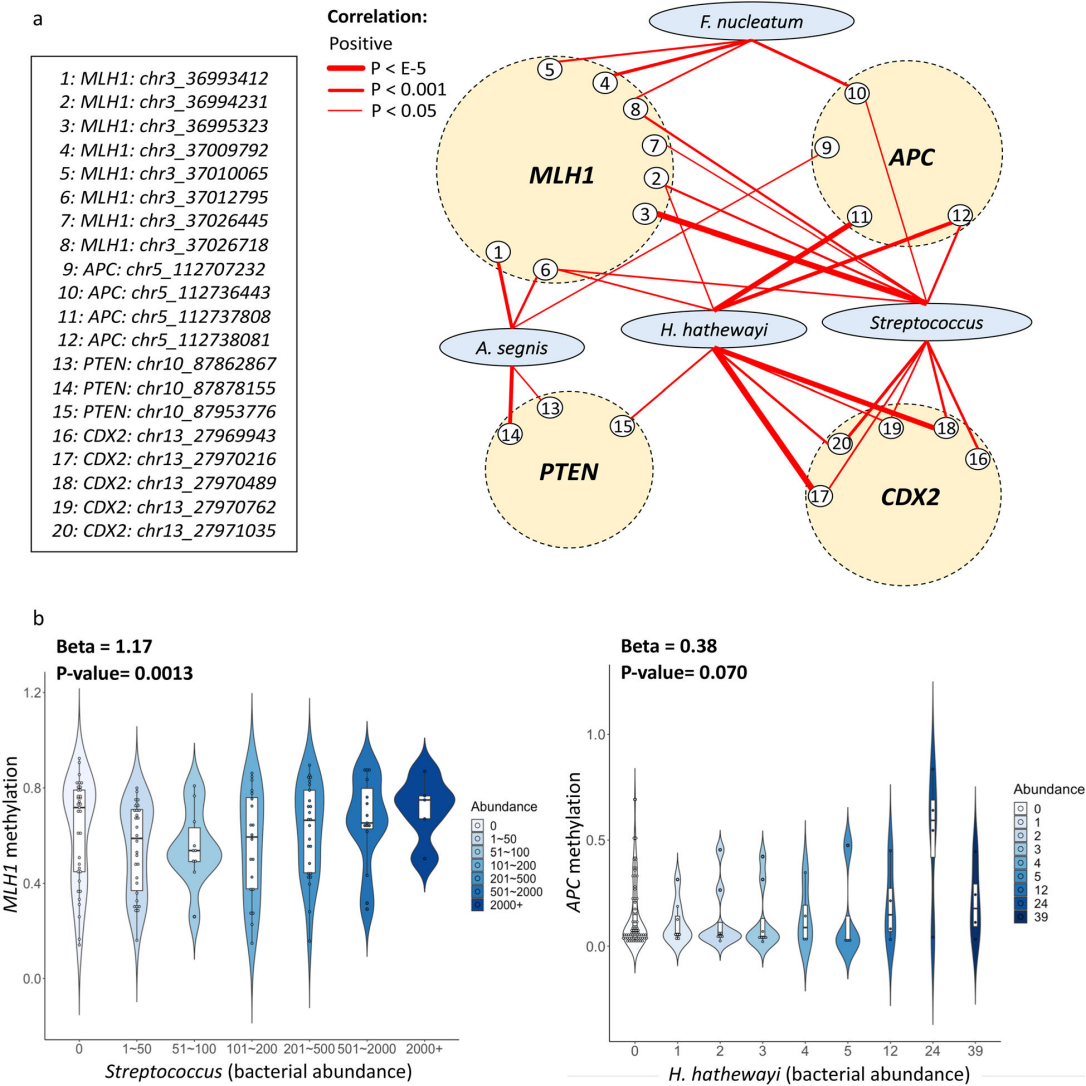
Bacteria interactions with target CRC TSGs. **a** Significant positive bacteria interactions involving *MLH1*, *APC*, *PTEN*, *P16*, *CDX1*, and *CDX2*. Line width = −log (*P*). **b** Left: Violin plot of *MLH1* methylation versus *Streptococcus spp*. Right: *APC* versus *H. hathewayi*. Methylation levels of both genes increased as the bacteria abundance elevated

*MLH1*, *APC*, *PTEN*, *P16*, and *CDX1/2* are *bona fide* TSGs whose promoter hypermethylation and tumor-suppressing functions in CRC have been experimentally verified [13]. To investigate whether the mucosal microbiome is associated with methylation profiles of these specific TSGs, we evaluated all bacteria for interactions with these key TSGs. In this regard, *MLH1*, *APC*, *PTEN*, and *CDX2* showed positive interactions with multiple CRC-enriched bacteria (Fig. 3). Particularly, strong positive correlations between methylation of multiple CpG sites in the promoter of *CDX2* and *H. hathewayi*, as well as *MLH1* and *Streptococcus* spp. were observed. Hypermethylation of the promoter of *APC* was also positively associated with *H. hathewayi* and *Streptococcus* spp. (Table S2). These results suggested that CRC-enriched bacteria might be a potential driver of hypermethylation in the promoter regions of key TSGs in CRC.

### *F. nucleatum* and *H. hathewayi* increased DNMT expression and activity in colonic cell lines

**Fig. 4:**
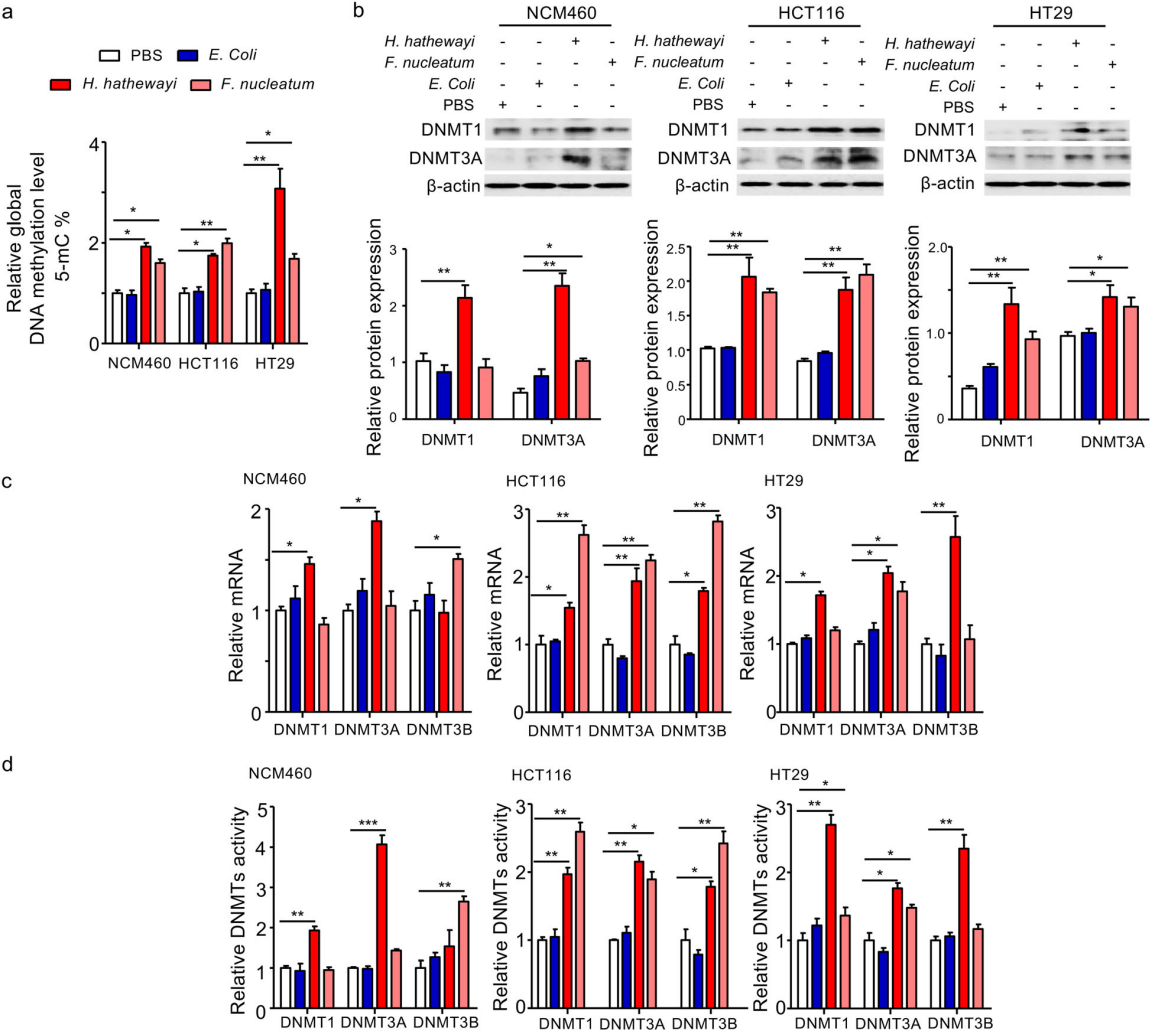
In vitro effects of *F. nucleatum* and *H. hathewayi* on DNMT expression and activity. **a***F. nucleatum* and *H. hathewayi* co-incubation increased the global DNA methylation (5-mC) levels in NCM460, HCT116, and HT29 cell lines. **b**–**c** RT-qPCR and Western blots confirmed that the mRNA and protein expression of DNMTs were positively regulated by *F. nucleatum* and *H. hathewayi*. **d** Nuclear DNMT3A activity was induced by *F. nucleatum* and *H. hathewayi*. Results in **a** to **d** show the average responses obtained from 3 independent experiments (*n* = 3 in each experiment). *P < 0.05; **P < 0.01;*** *P* < 0.001; significantly different between the indicated groups

Next, we investigated the downstream molecular mechanisms underlying the potential hypermethylation effects of *H. hathewayi* and *F. nucleatum* in CRC. Because DNMTs are a family of enzymes that initiate CpG methylation, we postulated that *F. nucleatum* and *H. hathewayi* might cause TSG promoter hypermethylation by regulating DNMT expression and activity. To confirm this, a colonic normal epithelial cell line (NCM460) and two colon CRC cell lines (HT29 and HCT116) were incubated with *F. nucleatum* and *H. hathewayi* for 4 h. We found that incubation with *F. nucleatum* and *H. hathewayi* led to significant increases in global DNA methylation (5-mC) levels in NCM460 (*p* < 0.05 for *H. hathewayi* and *p* < 0.05 for *F. nucleatum*), HCT116 (*p* < 0.05 for *H. hathewayi* and *p* < 0.01 for *F. nucleatum*) and HT29 (*p* < 0.01 for *H. hathewayi* and *p* < 0.05 for *F. nucleatum*) compared to PBS treatment or incubation with non-tumorigenic *Escherichia coli* MG1655 (Fig. 4a). Consistent with these observations, *F. nucleatum* and *H. hathewayi* induced the expression and nuclear activity of DNMT1 and DNMT3A in two CRC cell lines (HT29 and HCT116) (Fig. 4b–d). In the normal epithelial cell line NCM460, *H. hathewayi* also increased the expression and nuclear activity of DNMT1 and DNMT3A, whereas *F. nucleatum* induced mRNA expression and nuclear activity of DNMT3B (Fig. 4c and d).

### *H. hathewayi* promoted intestinal epithelial cell proliferation in conventional and germ-free mice and induced DNMT expression and activity in vivo

**Fig. 5:**
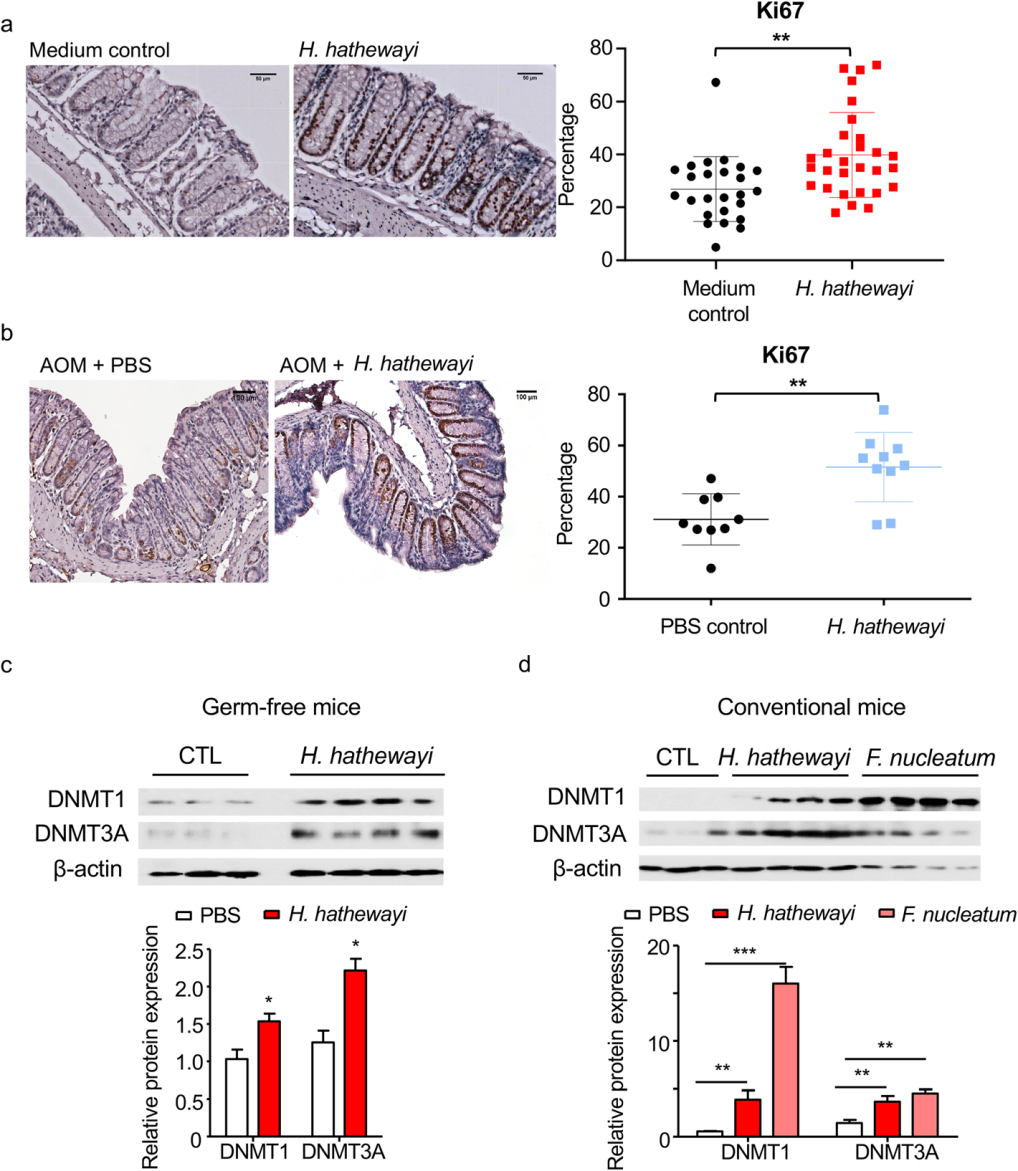
Effect of *H. hathewayi* on colonic epithelial cell proliferation in germ-free and AOM-injected conventional mice. **a** Immunohistochemistry showing Ki-67 positive cells in the colon of germ-free mice at 20 weeks after gavaging with *H*. *hathewayi*, and the relative proportion of Ki-67 positive cells. Scale bar: 50 μm. **b** Immunohistochemistry showing Ki-67 positive cells in the colon of AOM-injected, microbiota-depleted convectional mice at 6 weeks after bacterial gavage, and the relative proportion of Ki-67 positive cells. Scale bar: 100 μm. **C** Western blots showed that *H. hathewayi* increased the colonic protein expression of DNMT1 and DNMT3A in germ-free mice. **d** Western blots showed that *F. nucleatum* and *H. hathewayi* increased colonic protein expression of DNMT1 and DNMT3A in conventional mice. Each lane in **c** and **d** corresponds to a colonic sample harvested from an individual mouse. *P < 0.05; **P < 0.01 significantly different between the indicated groups

Our results indicated that upregulation of DNMT expression and activity was associated with *F. nucleatum-* and *H. hathewayi-*induced TSG promoter hypermethylation. *F. nucleatum* has been well characterized as a CRC-promoting bacterium [37], whereas the role of *H. hathewayi* in intestinal tumorigenesis remains unknown. To assess the potential tumor-promoting activity of *H. hathewayi*, we examined its in vivo effects in two separate mouse models, using germ-free and conventional specific pathogen-free (SPF) mice. Germ-free mice gavaged with *H. hathewayi* showed a significant increase in colonic Ki-67 immunoreactivity, when compared to control mice given PBS (*p* < 0.01) (Fig. 5a). Consistently, in conventional microbiota-depleted mice (using antibiotics) challenged with azoxymethane (AOM; a colon-specific chemical carcinogen) followed by *H*. *hathewayi* or PBS gavage, we observed significantly increased cell proliferation (*p* < 0.01) in the colon tissue of the *H*. *hathewayi* group as indicated by a higher proportion of Ki-67 positive cells (Fig. 5b).

In line with our in vitro findings, we observed an upregulation of DNMT1 and DNMT3A protein in *H. hathewayi*-inoculated conventional mice and germ-free mice as compared with corresponding untreated control mice (Fig. 5c and d). Meanwhile, we noted a similar effect in *F. nucleatum*-inoculated conventional mice (Fig. 5d) but not in *F. nucleatum*-inoculated germ-free mice (data not shown). These data collectively showed that enrichment of *F. nucleatum* or *H. hathewayi* in colonic mucosa activates DNMT expression and activity. Taken together, these results indicate that *H. hathewayi* could promote intestinal cell proliferation at least in part by modulating host methylation through inducing DNMT. Aside from DNMT, we observed that *H*. *hathewayi* could induce the mRNA expression of prostaglandin E_2_ biosynthetic pathway genes *Pla2g4c* and *Cox2* (Figure S4).

## Discussion

In the present study, through concurrent MBDCap-Seq and 16S rRNA gene sequencing from the same biopsies, we discovered novel associations between tumoral abundances of CRC-enriched bacteria and pervasive hypermethylation of TSG promoters in CRC and/or colorectal adenomas. This finding is consistent with a previous study by Sobhani et al*.* reporting that fecal microbiota transplantation from CRC patients, as compared to healthy controls, to germ-free mice induced more hypermethylated genes in the colonic mucosa [38]. To this end, our present study complemented their work by depicting the microbiome-methylome interaction at an unprecedented resolution by assessing the epigenome-wide effect of individual bacterial species and by pinpointing *F. nucleatum* and *H. hathewayi* as two key CRC-enriched bacteria mediating promoter hypermethylation.

Previous shotgun metagenomic studies from our group and other investigators have identified *F. nucleatum* and *H. hathewayi* as two of the most significantly enriched bacteria in stools and tissue biopsies of patients with CRC [7, 39]. *F. nucleatum* has already been identified as a driver bacterium to promote intestinal tumorigenesis through multiple mechanisms [19], including stimulation of the oncogenic Wnt/β-catenin signaling [9]. *H. hathewayi*, previously known as *Clostridium hathewayi*, is an anaerobic, endospore-forming Gram-negative bacillus that was first described in 2001 [40, 41]. Recent studies revealed that the mucosal abundance of *H. hathewayi* was positively correlated with the number of T helper 17 cells in the intestine [42] and was associated with the disease severity of Crohn’s disease [43]. Here, we for the first time characterized *H. hathewayi* as a potential CRC-promoting bacteria capable of increasing colonic epithelial cell proliferation in both conventional and germ-free mice. Nevertheless, it is noteworthy that colonic epithelial cell proliferation instead of the number of macroscopic tumors was used as a marker of cancer promotion in this study and our previous report [11]. We measured the histological outcomes at week 6 when macroscopic tumors had not yet formed in the AOM-injected conventional mouse model because we would like to assess the direct effect of *H. hathewayi*, which could not be done without effective depletion of the pre-existing gut microbiota. Since the microbiota-depleting effect of antibiotics could only last for weeks, outcomes at later time points were not measured to avoid the confounding effect of *H. hathewayi* on the gut microbiota composition. Future studies should therefore involve the use of carcinogen- or genetically-induced CRC in germ-free mice for a more comprehensive assessment of the pro-tumorigenic effect of *H. hathewayi*.

In mammals, there are three major DNMTs—DNMT1, DNMT3a, and DNMT3b. DNMT1 is responsible for maintenance DNA methylation activity needed for copying the methylation pattern during DNA replication, whereas DNMT3a and 3b mediate de novo DNA methylation which is essential for genome regulation [44]. In this study, *F. nucleatum* and *H. hathewayi* significantly increased the global DNA methylation (5-mC) levels in colonic epithelial cell lines NCM460, HCT116, and HT29. In keeping with this, *F. nucleatum* and *H. hathewayi* induced the expression and nuclear activity of DNMT1 and DNMT3A in two CRC cell lines (HT29 and HCT116). A consistent upregulation of DNMT1 and DNMT3a was confirmed in mouse colonic epithelium exposed to *H. hathewayi*. To this end, both DNMT1 and DNMT3a have been shown to be essential for the tumor-initiating ability of CRC stem-like cells [45, 46]. In a study on ulcerative colitis, a strong link between *Fusobacterium* enrichment and DNA methylation accumulation in the inflammatory colonic mucosa has been reported [47]. The mechanisms by which *F. nucleatum* and *H. hathewayi* promoted DNMT expression and subsequently triggered TSG promoter hypermethylation are presently unclear but higher expression of cyclooxygenase-2 (COX-2) and increased production of its product prostaglandin E_2_ might be involved. Moreover, upregulation of COX-2 has been documented in colonic tumors from mice inoculated with *F. nucleatum* [48], in which prostaglandin E_2_ could silence TSGs via DNMT-dependent DNA hypermethylation [49]. Consistently, we observed significant upregulation of two genes involved in prostaglandin E_2_ production, namely *Pla2g4a* (encoding phospholipase A2) and *Ptgs2* (encoding COX-2), in the colonic epithelium of mice gavaged with *H. hathewayi* (Figure S4). These findings suggested that *F. nucleatum* and *H. hathewayi* might epigenetically silence TSGs in a DNMT-dependent manner via the pro-inflammatory COX-2/prostaglandin E_2_ pathway. Aside from COX-2/prostaglandin E_2_, aberrant promoter hypermethylation of TSGs has been linked to other pro-inflammatory mediators. For instance, interleukin (IL)-1β directly induced promoter methylation of *CDH1* encoding the tumor suppressor E-cadherin in a mouse model of gastric cancer [50]. IL-6 also induced promoter hypermethylation of several important TSGs, namely *CHFR*, *GATA5*, and *PAX6*, in oral cancer cells [51]. Moreover, IL-8 was shown to increase the methylation of *CDH1* gene promoter in nasopharyngeal carcinoma cells by upregulating DNMT1 via the AKT pathway [52]. These studies collectively suggested that increased levels of pro-inflammatory cytokines could drive aberrant promoter hypermethylation of TSGs.

*APC* promoter hypermethylation is an early event in the classical adenoma-carcinoma sequence of the colon [53]. Gene-based analysis focusing on *APC* demonstrated that the abundances of a number of CRC-enriched bacteria, including *H. hathewayi*, *F. nucleatum*, and *Streptococcus* spp., exhibited positive correlation with *APC* promoter hypermethylation, suggesting that altered microbiota composition might contribute to the silencing of this prominent TSG. The loss of expression of *CDX2* which encodes a homeobox protein that maintains the intestinal phenotype is another common event in CRC and foreshadows poor clinical outcome [54]. In this study, the promoter hypermethylation of *CDX2* is strongly associated with *H. hathewayi*. Whether the mucosal abundance of *H. hathewayi* could have a similar prognostic significance as per *F. nucleatum*, however, warrants further study. *EYA4* is another TSG co-regulated by *F. nucleatum* and *H. hathewayi*. Aberrant silencing of *EYA4* has been shown to enhance the Wnt/β-catenin signaling during CRC development [55]. Collectively, our findings suggested that altered microbiota pathogens might contribute to the silencing of the prominent TSGs through inducing promoter hypermethylation. A notable finding of this study is that *F. nucleatum* and *H. hathewayi* were associated with promoter hypermethylation of different sets of TSGs. In this regard, it is known that the direct interaction with transcription factors dictates the genomic specificity of DNMT3a and DNMT3b [56]. It is therefore possible that *F. nucleatum* and *H. hathewayi* could invoke different sets of transcription factors to mediate respective targeted DNA methylation.

## Conclusions

We demonstrated for the first time that CRC-enriched bacteria, *F. nucleatum* and *H. hathewayi*, showed strong and pervasive correlations with TSG promoter hypermethylation in clinical specimens. These bacteria could directly enhance DNA methyltransferase activity in cell-line and/or animal models. Taken together, our comprehensive integrative analysis revealed the unexpected host epigenetic regulation of multiple TSG promoters by *F. nucleatum* and *H. hathewayi* in microbiota-driven intestinal tumorigenesis.

## Supplementary information

**Additional file 1: Figure S1.** Differential genome-wide coverage of MBDCap sequence reads. Data was arcsine-square-root-transformed to help visualize low coverage regions. Rows and columns represent chromosomal segments and sample methylation profiles, respectively. Row heights are normalized relative chromosome-wide CpG density. Boxes highlight significant tissue phenotypes that had the highest mean sequence coverage for a genomic feature within a chromosome. TPM, transcripts per million. Circles, P < 0.05; Stars, P < 0.01. P-values were from Kruskal-Wallis tests. **Figure S2.** Differentially methylated regions (DMRs) in colorectal carcinogenesis. Of the 1,059 representative DMRs identified, 465 (43.9%) were annotated with HGNCs; this subset of segments was assigned to promoter regions (96 or 20.6%) in which CpG islands were present (48 or 50%). Rows and columns represent genomic segments and sample DMR profiles, respectively. **Figure S3.** Heatmap of bacteria abundances in phenotype groups. From left to right the bacteria abundances are displayed in groups of normal, adenoma adjacent (AN), adenoma, CRC AN, and CRC. From top to bottom the bacteria are ranked by the fold-change between CRC and CRC AN samples. The bacteria shown are the ones with differential abundance between any two phenotype groups (P < 0.2). **Figure S4.** Induction of prostaglandin E2 biosynthetic pathway by H. hathewayi. Expression of Pla2g4c and Cox2 in colonic epithelium of mice gavaged with or without H. hathewayi was quantified by RT-qPCR. Expression levels were compared using t-test. *, P < 0.05; ** P < 0.01 significantly different between the indicated groups. **Supplementary Table 1.** Top bacteria-methylation interactions of C. hathewayi and F. nuleatum with tumor suppressor genes. **Supplementary Table 2.** The positive significant (ZINB p-value ≤ 0.05) interactions of MLH1, APC, PTEN, P16, CDX1 and CDX2 with bacteria.

## Data Availability

Complete datasets supporting the findings of this article are in the European Bioinformatics Institute (EBI)-European Nucleotide Archive (accession number: PRJEB35776).

## Abbreviations

5-mC: 5-Methylcytosine
COX-2: Cyclooxygenase-2
CRC: Colorectal cancer
DNMT: DNA methyltransferase
MBDCap-Seq: Methyl-CpG binding domain-based capture sequencing
TSG: Tumor-suppressor gene
