# Supplementary material for: Bacteria pathogens drive host colonic epithelial cell promoter hypermethylation of tumor suppressor genes in colorectal cancer
Source: Microbiome. 2020 Jul 16;8:108. doi: 10.1186/s40168-020-00847-4 (PMC7367367; doi:10.1186/s40168-020-00847-4)
Supplement: Supplementary file 2 — Additional file 1: Figure S1. Differential genome-wide coverage of MBDCap sequence reads. Data was arcsine-square-root-transformed to help visualize low coverage regions. Rows and columns represent chromosomal segments and sample methylation profiles, respectively. Row heights are normalized relative chromosome-wide CpG density. Boxes highlight significant tissue phenotypes that had the highest mean sequence coverage for a genomic feature within a chromosome. TPM, transcripts per million. Circles, P < 0.05; Stars, P < 0.01. P-values were from Kruskal-Wallis tests. Figure S2. Differentially methylated regions (DMRs) in colorectal carcinogenesis. Of the 1,059 representative DMRs identified, 465 (43.9%) were annotated with HGNCs; this subset of segments was assigned to promoter regions (96 or 20.6%) in which CpG islands were present (48 or 50%). Rows and columns represent genomic segments and sample DMR profiles, respectively. Figure S3. Heatmap of bacteria abundances in phenotype groups. From left to right the bacteria abundances are displayed in groups of normal, adenoma adjacent (AN), adenoma, CRC AN, and CRC. From top to bottom the bacteria are ranked by the fold-change between CRC and CRC AN samples. The bacteria shown are the ones with differential abundance between any two phenotype groups (P < 0.2). Figure S4. Induction of prostaglandin E2 biosynthetic pathway by H. hathewayi. Expression of Pla2g4c and Cox2 in colonic epithelium of mice gavaged with or without H. hathewayi was quantified by RT-qPCR. Expression levels were compared using t-test. *, P < 0.05; ** P < 0.01 significantly different between the indicated groups. Supplementary Table 1. Top bacteria-methylation interactions of C. hathewayi and F. nuleatum with tumor suppressor genes. Supplementary Table 2. The positive significant (ZINB p-value ≤ 0.05) interactions of MLH1, APC, PTEN, P16, CDX1 and CDX2 with bacteria. [file 40168_2020_847_MOESM1_ESM.pdf]

SUPPLEMENTARY FIGURE LEGENDS

Figure S1 Differential genome-wide coverage of MBDCap sequence reads. Data was arcsine-square-root-transformed to help visualize low coverage regions. Rows and columns represent chromosomal segments and sample methylation profiles, respectively. Row heights are normalized relative chromosome-wide CpG density. Boxes highlight significant tissue phenotypes that had the highest mean sequence coverage for a genomic feature within a chromosome. TPM, transcripts per million. Circles, $P < 0.05$; Stars, $P < 0.01$. P-values were from Kruskal-Wallis tests.

Figure S2 Differentially methylated regions (DMRs) in colorectal carcinogenesis. Of the 1,059 representative DMRs identified, 465 (43.9%) were annotated with HGNCs; this subset of segments was assigned to promoter regions (96 or 20.6%) in which CpG islands were present (48 or 50%). Rows and columns represent genomic segments and sample DMR profiles, respectively.

Figure S3 Heatmap of bacteria abundances in phenotype groups. From left to right the bacteria abundances are displayed in groups of normal, adenoma adjacent (AN), adenoma, CRC AN, and CRC. From top to bottom the bacteria are ranked by the fold-change between CRC and CRC AN samples. The bacteria shown are the ones with differential abundance between any two phenotype groups ($P < 0.2$).

Figure S4 Induction of prostaglandin E_2 biosynthetic pathway by *H. hathewayi*. Expression of *Pla2g4c* and *Cox2* in colonic epithelium of mice gavaged with or without *H. hathewayi* was quantified by RT-qPCR. Expression levels were compared using *t*-test. *, $P < 0.05$; ** $P < 0.01$ significantly different between the indicated groups.

Figure S1

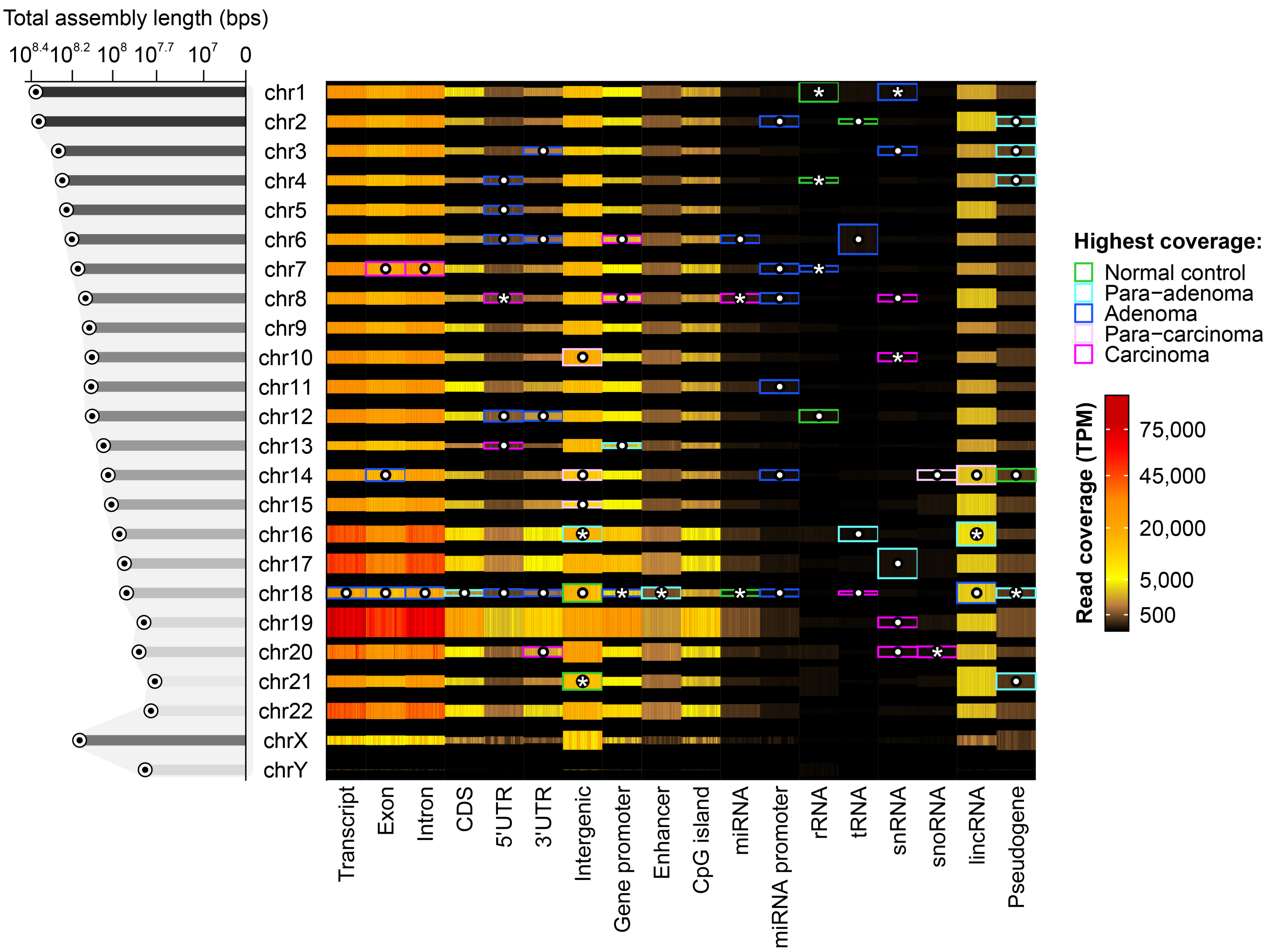

Total assembly length (bps)
$10^{8.4}$ $10^{8.2}$ 10^{8} $10^{7.7}$ 10^{7} 0
chr1
chr2
chr3
chr4
chr5
chr6
chr7
chr8
chr9
chr10
chr11
chr12
chr13
chr14
chr15
chr16
chr17
chr18
chr19
chr20
chr21
chr22
chrX
chrY
Transcript
Exon
Intron
CDS
5'UTR
3'UTR
Intergenic
Gene promoter
Enhancer
CpG island
miRNA
miRNA promoter
rRNA
tRNA
snRNA
snoRNA
lincRNA
Pseudogene
Highest coverage:
Normal control
Para−adenoma
Adenoma
Para−carcinoma
Carcinoma
Read coverage (TPM)
75,000
45,000
20,000
5,000
500

Figure S2

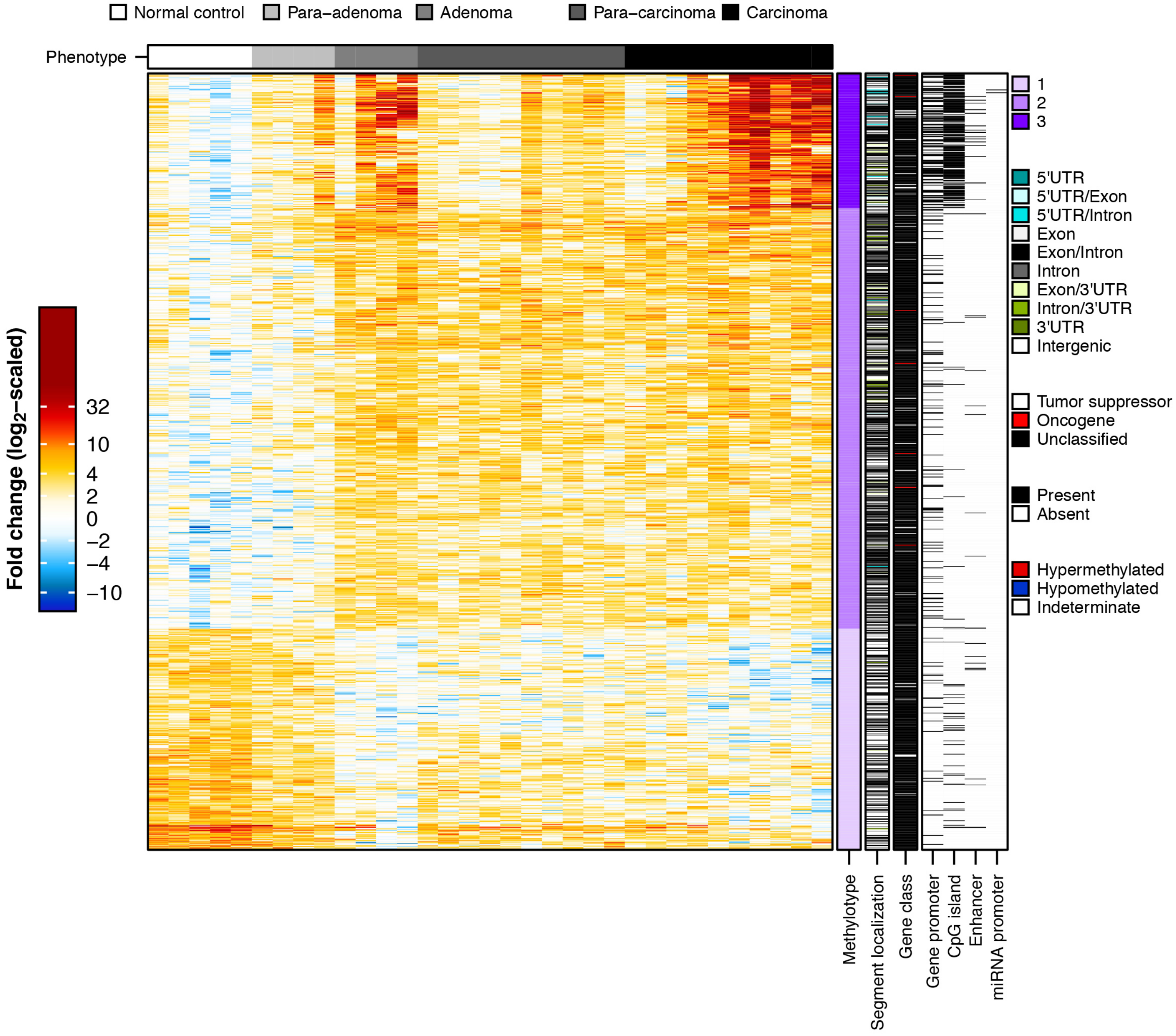

Normal control
Para−adenoma
Adenoma
Para−carcinoma
Carcinoma
Phenotype
Fold change (log2−scaled)
32
10
4
2
0
−2
−4
−10
1
2
3
5'UTR
5'UTR/Exon
5'UTR/Intron
Exon
Exon/Intron
Intron
Exon/3'UTR
Intron/3'UTR
3'UTR
Intergenic
Tumor suppressor
Oncogene
Unclassified
Present
Absent
Hypermethylated
Hypomethylated
Indeterminate
Methylotype
Segment localization
Gene class
Gene promoter
CpG island
Enhancer
miRNA promoter

Figure S3

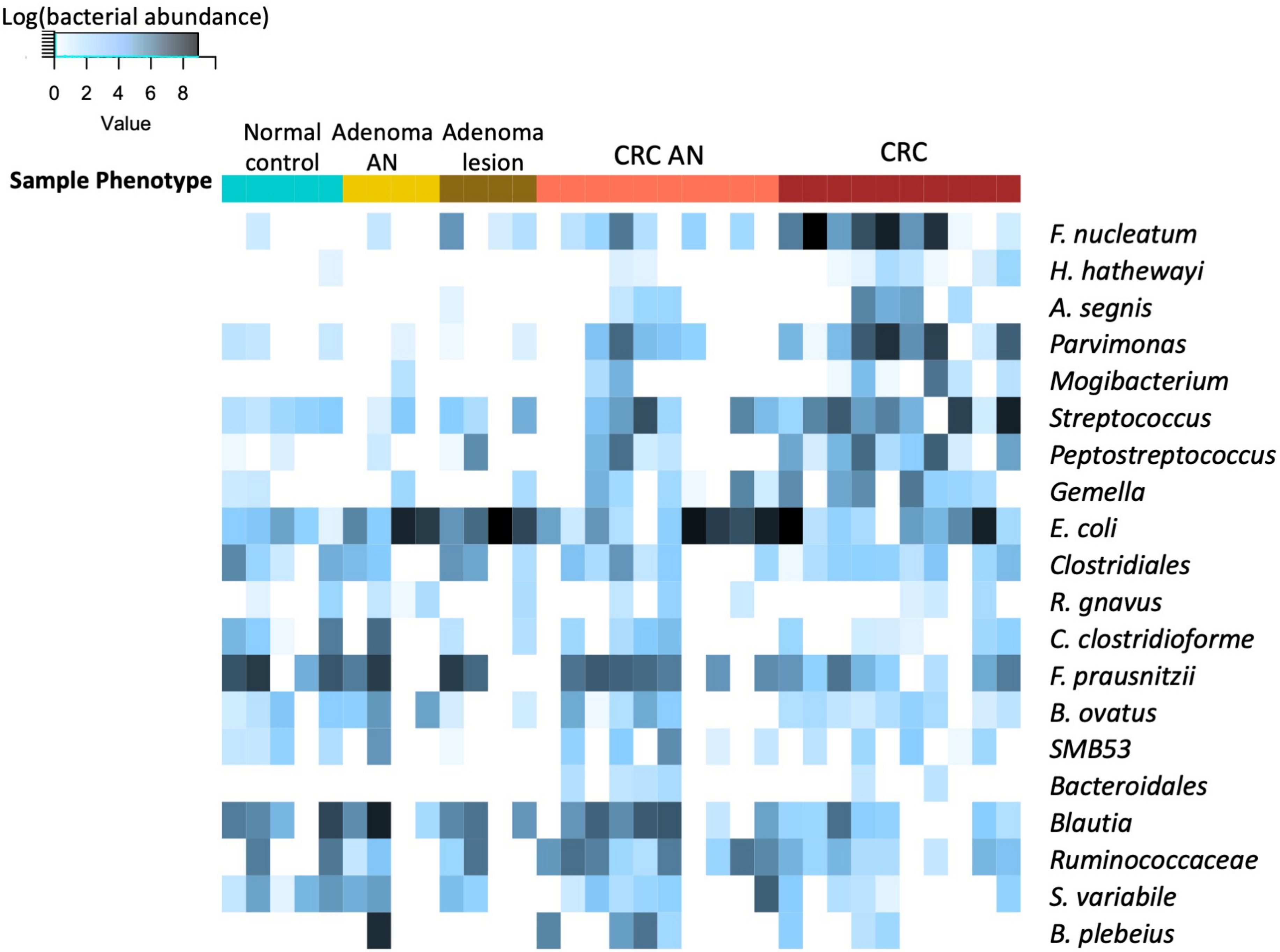

Log(bacterial abundance)
0 2 4 6 8
Value
Sample Phenotype
Normal control
Adenoma AN
Adenoma lesion
CRC AN
CRC
F. nucleatum
H. hathewayi
A. segnis
Parvimonas
Mogibacterium
Streptococcus
Peptostreptococcus
Gemella
E. coli
Clostridiales
R. gnavus
C. clostridioforme
F. prausnitzii
B. ovatus
SMB53
Bacteroidales
Blautia
Ruminococcaceae
S. variabile
B. plebeius

Figure S4

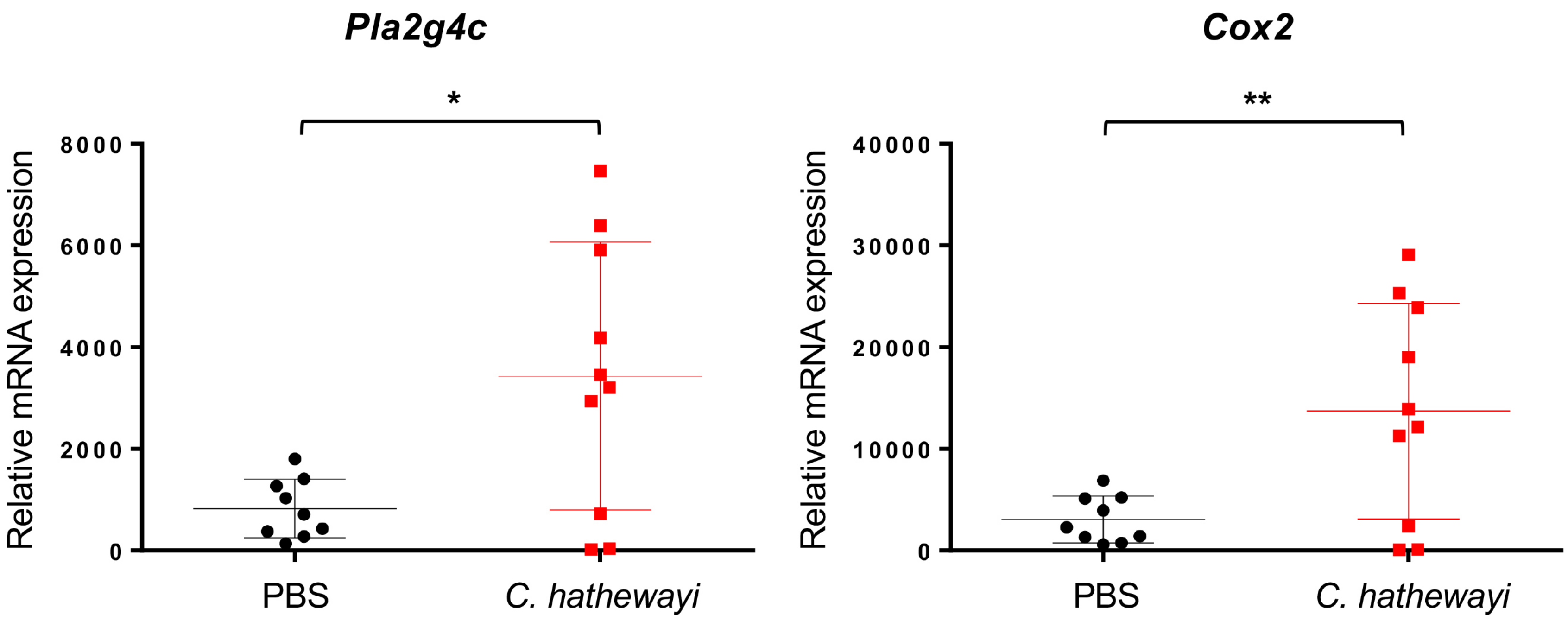

Pla2g4c
*
Relative mRNA expression
8000
6000
4000
2000
0
PBS
C. hathewayi
Cox2
**
Relative mRNA expression
40000
30000
20000
10000
0
PBS
C. hathewayi

Supplementary Table 1. Top bacteria-methylation interactions of *C. hathewayi* and *F. nuleatum* with tumor suppressor genes.

Bacteria	Methylation Segment	Gene	ZINB[1] Beta	Beta P-value	Spearman Rho	Rho P-value[2]
C. hathewayi	chr2_5693962	***SOX11***	4.22	1.71E-05	0.38	0.030
	chr20_23049391	***THBD***	1.43	6.77E-07	0.11	0.029
	chr4_153788818	***SFRP2***	1.57	1.16E-05	0.38	0.029
	chr20_62475232	***GATA5***	1.46	9.52E-05	0.17	0.030
	chr6_133240654	***EYA4***	1.12	3.85E-05	0.22	0.119
	chr6_151807657	***ESR1***	1.33	2.50E-04	0.17	0.103
F. nucleatum	chr8_124562803	***MTSS1***	6.47	1.54E-06	0.44	0.011
	chr20_57393064	***RBM38***	3.45	6.35E-05	0.48	0.005
	chr20_57392518	***RBM38***	1.95	1.93E-06	0.44	0.010
	chr16_2107561	***PKD1***	7.56	6.47E-04	0.48	0.005
	chr6_133242292	***EYA4***	2.19	5.59E-04	0.44	0.010
	chr6_133241200	***EYA4***	1.53	3.98E-04	0.47	0.006
	chr20_43189420	***PTPRT***	1.96	3.79E-04	0.39	0.026

1. ZINB: Zero-Inflated Negative Binomial Regression. ZINB Beta: the beta in the count model of ZINB. *P*-value threshold < 0.001.
2. Spearman rank correlation test. *P*-value threshold < 0.15.

Supplementary Table 2. The positive significant (ZINB *p*-value ≤ 0.05) interactions of *MLH1*, *APC*, *PTEN*, *P16*, *CDX1* and *CDX2* with bacteria.

Gene	*Methylation Segment*	*Bacteria*	**ZINB Beta**	**ZINB P-value**	**Spearman Rho**	**Rho P-value**
MLH1	*chr3_36995323*	***Streptococcus.spp***	10.01	4.41E-06	0.24	0.19
	chr3_36993412	***A. segnis***	2.12	9.29E-04	0.08	0.64
	chr3_37009792	***F. nucleatum***	3.45	1.22E-03	0.29	0.10
	chr3_37026718	***Streptococcus.spp***	5.48	7.96E-03	0.03	0.87
	chr3_37026991	***C. clostridioforme***	18.10	1.01E-02	0.21	0.24
	chr3_36994231	***Streptococcus.spp***	1.65	1.08E-02	0.28	0.12
	chr3_37010065	***F. nucleatum***	6.54	1.40E-02	0.15	0.42
	chr3_36993685	***Ruminococcaceae***	2.21	2.17E-02	0.32	0.07
	chr3_37026718	***F. nucleatum***	7.21	2.38E-02	0.04	0.81
	chr3_37026445	***C. clostridioforme***	3.53	2.67E-02	0.25	0.16
	chr3_37012795	***C. hathewayi***	6.55	2.69E-02	0.10	0.56
	chr3_37010611	***Ruminococcaceae***	4.64	3.35E-02	0.09	0.62
	chr3_36993412	***Ruminococcaceae***	1.89	3.68E-02	0.24	0.18
	chr3_36995050	***B. plebeius***	6.89	4.26E-02	0.33	0.06
	chr3_36995323	***Parvimonas.spp***	6.94	4.53E-02	0.06	0.74
	chr3_36994231	***C. hathewayi***	2.11	4.60E-02	0.06	0.75
	chr3_37026445	***Streptococcus.spp***	1.63	4.98E-02	0.07	0.70
APC	*chr5_112737808*	***C. hathewayi***	1.11	2.26E-05	0.11	0.54
	chr5_112736443	***B. plebeius***	7.69	2.29E-04	0.27	0.14
	chr5_112738081	***C. hathewayi***	4.74	3.14E-04	0.20	0.28
	chr5_112707232	***A. segnis***	3.23	3.98E-04	0.11	0.53
	chr5_112778485	***C. clostridioforme***	2.69	6.71E-04	0.05	0.80
	chr5_112825441	***Peptostreptococcus***	4.91	1.06E-03	0.09	0.60
	chr5_112778485	***B. plebeius***	3.34	1.75E-03	0.25	0.16
	chr5_112736443	***F. nucleatum***	3.95	2.73E-03	0.19	0.28
	chr5_112707232	***Ruminococcaceae***	1.58	4.93E-03	0.10	0.60
	chr5_112738081	***Streptococcus.spp***	2.67	8.20E-03	0.14	0.43
	chr5_112738081	***Parvimonas.spp***	3.67	1.02E-02	0.29	0.10

	chr5_112736443	***Streptococcus.spp***	2.44	4.81E-02	0.09	0.60
PTEN	*chr10_87953776*	***C. hathewayi***	4.80	1.96E-02	0.24	0.17
	chr10_87878155	***A. segnis***	4.16	4.59E-02	0.14	0.44
	chr10_87862867	***A. segnis***	3.97	4.61E-02	0.14	0.44
CDX2	*chr13_27970216*	***C. hathewayi***	2.18	3.54E-06	0.18	0.31
	chr13_27966667	***C. clostridioforme***	1.59	4.61E-06	0.03	0.88
	chr13_27970489	***C. hathewayi***	1.35	1.88E-05	0.12	0.49
	chr13_27966121	***Ruminococcaceae***	1.98	2.66E-05	0.25	0.16
	chr13_27966667	***S. variabile***	1.20	1.41E-04	0.26	0.15
	chr13_27967486	***S. variabile***	2.52	2.08E-04	0.22	0.21
	chr13_27969397	***S. variabile***	1.90	2.72E-04	0.26	0.14
	chr13_27967759	***C. clostridioforme***	2.11	2.98E-04	0.11	0.55
	chr13_27970762	***C. clostridioforme***	3.11	4.17E-04	0.19	0.28
	chr13_27968032	***S. variabile***	1.16	5.68E-04	0.29	0.10
	chr13_27965575	***Mogibacterium***	7.07	1.44E-03	0.10	0.58
	chr13_27969670	***S. variabile***	1.61	1.62E-03	0.20	0.28
	chr13_27971035	***Streptococcus.spp***	0.98	1.82E-03	0.34	0.05
	chr13_27966940	***S. variabile***	1.30	1.98E-03	0.22	0.22
	chr13_27969397	***C. clostridioforme***	1.59	2.11E-03	0.02	0.90
	chr13_27965302	***C. clostridioforme***	5.72	2.89E-03	0.09	0.61
	chr13_27967759	***S. variabile***	1.93	3.25E-03	0.11	0.54
	chr13_27969943	***Streptococcus.spp***	2.42	4.99E-03	0.24	0.19
	chr13_27968851	***Ruminococcaceae***	1.39	5.88E-03	0.34	0.05
	chr13_27970489	***Streptococcus.spp***	0.70	6.25E-03	0.20	0.27
	chr13_27966394	***C. clostridioforme***	1.17	7.21E-03	0.35	0.05
	chr13_27971035	***C. hathewayi***	1.11	7.27E-03	0.11	0.55
	chr13_27966394	***S. variabile***	1.36	8.96E-03	0.25	0.16
	chr13_27968851	***S. variabile***	1.38	9.75E-03	0.34	0.05
	chr13_27967759	***B. plebeius***	2.33	1.14E-02	0.11	0.53
	chr13_27966667	***B. plebeius***	1.50	1.36E-02	0.12	0.51
	chr13_27968578	***Ruminococcaceae***	2.03	1.63E-02	0.35	0.05
	chr13_27965848	***C. clostridioforme***	1.70	2.24E-02	0.45	0.01

chr13_27969943	***S. variabile***	1.85	2.33E-02	0.15	0.40
chr13_27966394	***B. plebeius***	1.59	2.35E-02	0.23	0.20
chr13_27970216	***Streptococcus.spp***	0.87	2.36E-02	0.15	0.39
chr13_27967213	***S. variabile***	1.91	2.78E-02	0.05	0.79
chr13_27967759	***B. ovatus***	1.45	2.84E-02	0.04	0.80
chr13_27970762	***Streptococcus.spp***	1.26	3.13E-02	0.03	0.85
chr13_27965302	***S. variabile***	2.92	3.42E-02	0.46	0.01
chr13_27968305	***S. variabile***	0.85	3.81E-02	0.31	0.08
chr13_27966121	***S. variabile***	1.40	3.85E-02	0.13	0.47
chr13_27968305	***Ruminococcaceae***	0.92	4.17E-02	0.31	0.08
chr13_27967486	***B. plebeius***	2.05	4.72E-02	0.03	0.87

1. ZINB: Zero-Inflated Negative Binomial Regression. ZINB Beta: the beta in the count model of ZINB. *P*-value threshold < 0.05.
